# Frailty and quality of life in older ICU survivors: a scoping review of assessment tools and methodologies

**DOI:** 10.1186/s12877-026-07104-7

**Published:** 2026-02-10

**Authors:** Gunhild Kjaergaard-Andersen, Rajesh Prabhakar Bhavsar, Hanne Irene Jensen, Linda Juel Ahrenfeldt, Niels Christian Hvidt, Thomas Strøm

**Affiliations:** 1 Department of Anaesthesiology and Intensive Care Department, Hospital Sonderjylland, University Hospital of Southern Denmark, Odense, Denmark; 2 Department of Regional Health Research, University of Southern Denmark, Odense, Denmark; 3 Department of Anaesthesiology and Intensive Care, Lillebaelt Hospital, University Hospital of Southern Denmark, Odense, Denmark; 4 Research Unit of General Practice, Department of Public Health, University of Southern Denmark, Esbjerg-Odense, Denmark; 5 Research Unit for General Practice, Department of Public Health, University of Southern Denmark, Odense, Denmark; 6 Academy for Geriatric Cancer Research, Odense University Hospital, Odense, Denmark; 7 Department of Intensive Care, Odense University Hospital, Odense, Denmark; 8 Department of Anaesthesiology and Intensive Care, Hospital Soenderjylland, Kresten Philipsens vej 15, Aabenraa, 6200 Denmark

**Keywords:** Elderly frail, Geriatric, Intensive care unit, Quality of life.

## Abstract

**Background:**

Life expectancy increases, leading to growth in an older population where frailty and comorbidities are more prevalent. Subsequent increased intensive care unit (ICU) treatment results in prolonged rehabilitation and post-discharge loss of physiological and intellectual functions, negatively influencing quality of life (QoL).

**Methods:**

This scoping review aims to identify existing research on assessment methods for frailty, QoL, and health-related outcomes for these ICU survivors.

The review followed Joanna Briggs Institute guidelines for scoping reviews and reported using the PRISMA-P checklist.

Systematic searches were conducted in Embase, Medline, PsycINFO, and CINAHL (2013 to 2023). Included studies were primary, peer-reviewed, or empirical studies written in English or Scandinavian, patients aged 70+, admitted to ICU more than 48 h, and inclusion of both frailty and QoL. A librarian qualified the search. Two reviewers independently screened articles and extracted data; a third reviewer resolved discrepancies.

**Results:**

A total of 5,198 articles were identified. Nine met the eligibility criteria. Different tools assessed frailty and QoL. For frailty, Clinical Frailty Scale, Karnofsky Performance Status, Modified Katz Index of Activities of Daily Living, Groningen Frailty Indicator, CGA questionnaire, or Barthel Index were used. A combination of scales (SF-12, SF-36, EQ-5D, EQ-5D-5 L, or EQ-5D-3 L), questionnaires, or interviews (regarding residential and functional status and self-reported health-related QoL) was used for QoL.

**Conclusion:**

Frail older ICU survivors experience persistent physical, cognitive, and symptom-related challenges poorly captured by conventional QoL assessments, particularly regarding psychological and subjective experiences. The few studies addressing both frailty and QoL make it difficult to establish evidence, highlighting the need for standardised, multidimensional, patient-centred frameworks.

**Trial registration:**

Not applicable.

**Supplementary Information:**

The online version contains supplementary material available at 10.1186/s12877-026-07104-7.

## Introduction

Advancements in medical technology and treatments have significantly increased life expectancy [1]. As individuals age, their risk of developing comorbidities increases [2]. Although frailty and age are not always correlated, older adults with multiple comorbidities are especially vulnerable to the effects of diseases and ageing [3].

Frailty is commonly defined as a syndrome characterised by increased vulnerability and deteriorated physical functions, leading to adverse health outcomes and heightened mortality [4]. It is influenced by multiple domains, including social, physical, cultural, and environmental factors [5]. Frail individuals receiving advanced medical treatments often experience hospitalisation, with a considerable number requiring intensive care unit (ICU) care [6, 7].

ICU stays can exacerbate physical and cognitive impairments, prolonging the rehabilitation period after discharge [8–10]. In some cases, these impairments are irreversible, significantly affecting the patient’s quality of life (QoL) [7]. Among the elderly, reduced resilience diminished functional capacity, and difficulties coping with stress further limit recovery from critical illness [11, 12]. These challenges can lead to increased fear of falling, reduced ability to perform basic daily activities, and a subsequent decline in QoL [13].

QoL is a multifaceted and subjective concept influenced by factors such as life perception, spirituality, mental and physical health, and social, cultural, and economic circumstances [14, 15].

These complexities underscore the need for effective assessment tools that capture the multidimensional nature of frailty and QoL, particularly in frail elderly ICU patients.

Despite the growing recognition of frailty and QoL as critical determinants of health outcomes, a gap exists in the current literature. Specifically, there is a lack of consistency in the methods used to assess these factors among frail elderly patients following ICU discharge. This inconsistency not only limits the ability to compare findings across studies but also hampers the development of targeted interventions and policies to improve outcomes for this vulnerable population. Assessing frailty and QoL concurrently allows for a more holistic understanding of how biological vulnerability translates into patient-centred outcomes. This integrated approach is essential in geriatric and critical care research, as it captures the multidimensional impact of frailty on both clinical prognosis and the lived experience of older adults [16–18].

Given these challenges, this scoping review aims to map and critically evaluate the existing methods for assessing both frailty and QoL in older ICU patients.

A scoping review is particularly well-suited to this purpose, as it enables a comprehensive mapping of the available evidence and captures the heterogeneity in frailty and QoL assessment methods across studies. Moreover, this approach facilitates the identification of methodological inconsistencies and evidence gaps. By highlighting these gaps, the review aims to provide a foundation for future research and support the development of standardised assessment frameworks that more effectively address the unique needs of this patient population.

## Objectives

This scoping review aims to map the current evidence on assessment methods for both frailty and QoL for frail older ICU survivors, identifying research gaps and highlighting areas for future studies. Here, we specifically focus on identifying the tools used to assess frailty and QoL, determining the health-related variables employed in defining frailty and QoL, and exploring frail, older patients’ perspectives on QoL post-ICU discharge.

## Methods

### Protocol and registration

The protocol for this scoping review is published in BMJ Open [19].

The protocol is reported by the adapted Preferred Reporting Items for Systematic Reviews and Meta-Analyses Protocols (PRISMA-P) checklist tailored explicitly for scoping reviews [20, 21].

This scoping review was conducted after the Joanna Briggs Institute (JBI) guidance for scoping reviews [20].

### Eligibility criteria

The eligible studies were primary, peer-reviewed, or empirical studies focusing on patients older than 70 years. The studies needed to include more than 50% of patients 70 years and above, to ensure that the studies predominantly reflected the target group. The patients should be admitted to all kinds of ICUs worldwide and discharged from the ICU after a stay exceeding 48 h. The age threshold of 70 was selected based on the likelihood of individuals remaining active in the workforce despite increasing age [22]. The studies investigating both frailty and QoL as primary concepts were considered. To maintain linguistic accessibility, studies were limited to those published in English, Danish, Norwegian or Swedish between 2013 and 2023. Exclusion criteria were studies focusing on postoperative patients staying in ICU for less than 48 h, no mention of frailty and QoL, Abstract-only studies were excluded after failing to attempt to obtain the full text. Due to variable review standards, conference papers and grey literature were not included. Doctoral theses were excluded, as they generally compile already-published in peer-reviewed studies.

Inclusion and exclusion criteria are detailed in Table 1.

**Table 1:** Inclusion and exclusion criteria for this scoping review on the assessment of QoL for frail, older patients’ post-ICU discharge

Characteristics of studies	Inclusion criteria	Exclusion criteria
Age of patients	70 years and above: For studies that involve both patients under and above 70 years, the studies were included if the percentage of patients aged 70 + years exceeded 50%	
Type of patients		Postoperative patients with less than 48 h of admission to the ICU
Duration of admission to the ICU	More than 48 h of admission to the ICU	
Levels of ICUs	All	
Location	Worldwide	
Language	English or Scandinavian	
Time frame	Published 2013–2023	
Type of Study	Primary, peer-reviewed, empirical studies	Abstracts, conference papers, and doctoral theses
Frailty in studies	Any measurement, proxy or description of frailty, regardless of definition	No measurement of frailty is employed.
Type of perspective	Patient perspectives relating to QoL post-ICU discharge	

### Search strategy

The first author (GKA) systematically searched four databases – Embase, MEDLINE, PsycINFO (via Ovid), and CINAHL (via EBSCO). The Cochrane Database of Systematic Reviews was searched for reviews relevant to our review question and used for additional search, by including only peer-reviewed research to ensure quality in the final synthesis.

The search strategy was developed in three steps: Population, Concept, and Context method, as described in detail in the protocol and according to JBI guidance [19, 20].

Because QoL and frailty are not strictly defined, an exhaustive search string from the following review questions was used: Which tools are used to assess and quantify QoL and frailty in frail, elderly patients’ post-ICU discharge, which health-related variables are used to define frailty and QoL, including frail and elderly patients’ perspectives on QoL post-ICU discharge. To ensure comprehensiveness and accuracy, an experienced information specialist and a librarian supported and qualified the search string Additional file 1.

A search using all identified MESH terms and keywords such as frailty, frail elderly, geriatric, aged, octogenarians, nonagenarians, health impairment, Intensive Care Unit, shock, sepsis, critical illness, survivors, hospital discharge, patient-reported outcomes, patient outcome assessment, Quality of Life, activities of daily living, satisfaction, synonyms, and index terms were undertaken across all included databases. All key terms were combined using Boolean operators AND/OR.

The initial search was performed in November 2023 and updated in April 2024 to ensure completeness.

### Selection of sources of evidence

All articles were first imported into EndNote to create a database for managing the references and then exported to the web-based reference program Covidence. Covidence was first used to remove duplicates and for screening. The screening process was conducted afterwards by two individual researchers (GKA and RPB). The initial screening focused on the titles and abstracts, followed by a thorough review of the filtered studies in their full-text form. In instances of disagreement, discussions were held to ensure a comprehensive and unbiased selection process. A third reviewer (TS) was consulted to resolve any remaining conflicts for a final decision. Given the limited number of studies identified in the first round, the systematic screening process was conducted twice to ensure that all relevant articles were thoroughly considered.

**Fig. 1:**
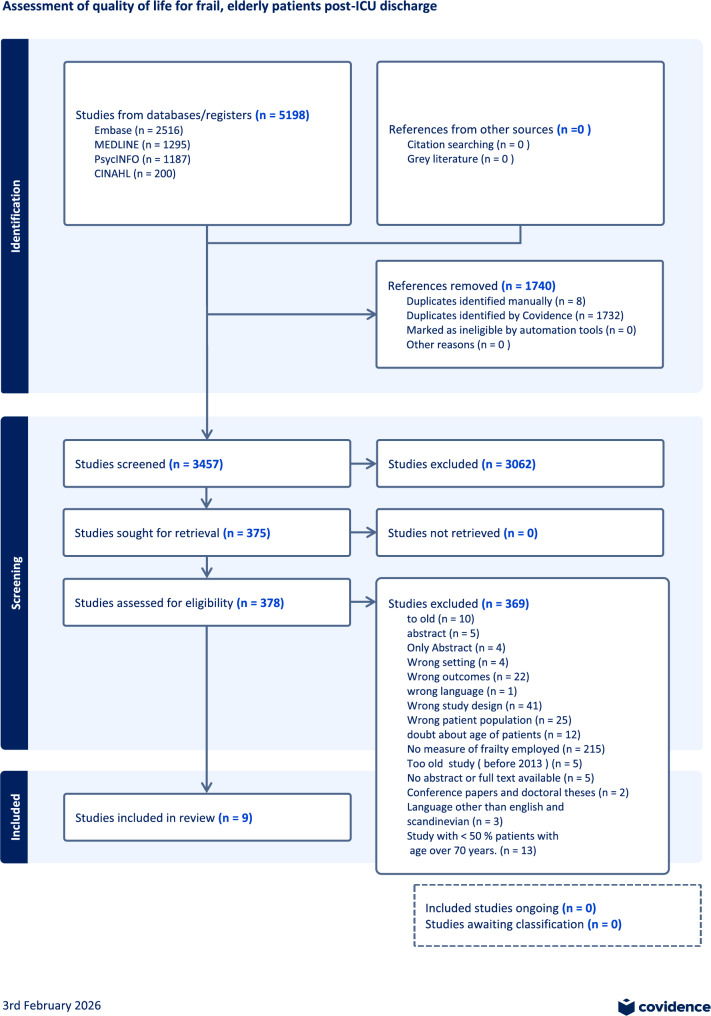
PRISMA-P flow diagram

The search results and the study inclusion and exclusion process are presented in a PRISMA-P flow diagram (Fig. 1).

### Data extraction

Two independent researchers (GKA and RPB) extracted data from the included studies using a standardised template in Covidence. In case of disagreements, the researchers discussed them for consensus and filled out the Covidence template for data extraction. Before initiating the analytical process, GKA and RPB critically reviewed and discussed the included studies.

Consistent with guidance on conducting scoping reviews, this review did not include an appraisal of the methodological quality or risk of bias [23].

### Data items

The extracted data encompassed study characteristics such as title, first author, year of publication, country of origin, study design, patient demographics (age, sex ratio - for assessing whether the findings are representative of the broader population, and sample size), follow-up duration, and specific tools for assessing frailty and QoL. Emphasis was placed on the methods utilised to evaluate frailty, QoL, and health-related outcome measures.

Concerning the number of included patients, we noted the number of patients who participated in the study by filling out a survey and/or participating in an interview, because only this part of the population was of interest for this scoping review. Due to the size of the data extraction table, it has been divided into two parts.

#### Use of AI

After the article had been written, AI programs (Grammarly, ChatGPT, and Gemini) were used to qualify the text as more fluent and congruent for reading.

## Results

A total of 5,198 articles were identified from the electronic databases: Embase (2,516), MEDLINE (1,295), PsycINFO (1,187), and CINAHL (1,732). Covidence identified and removed duplicates, and eight duplicates were identified and removed manually, leaving 3,457 articles for screening. Title and abstract screening excluded 3,062 studies, leaving 378 articles eligible for full-text assessment that needed to be considered for inclusion. Of these, 369 studies were excluded based on the original exclusion criteria. The primary reasons for exclusion were: No mention of measures of frailty (217), wrong patient population (45), different study design (41), wrong outcomes (20), too old studies - published before 2013 (15), doubt about the age of patients (12), only abstracts (9), different methodological settings (4), written in wrong language (4) conference papers and doctoral theses (2). No new studies were found after the repeated, systematic screening. Thus, nine studies remained eligible for final assessment, as shown in the PRISMA-P flowchart Fig. 1.

### Characteristics of sources of evidence

The nine included studies originated from different countries: Canada [24, 25], Belgium [26, 27], the Netherlands [28], pan-European [29], China [30], the United States [31], and Spain [32]. Two were retrospective, while seven were prospective observational studies. Four multicenter studies were conducted across multiple hospitals or research centres, with participation from several locations. Five were single-centre studies. All the multicenter studies were prospective studies. The number of patients included in the studies ranged from 48 to 717, with a female-to-male ratio between 23% and 48%. In the included studies, a substantial dropout rate was observed (Table 2).

**Table 2:** Data extraction: characteristics of the included studies

Title, first author, Year of publication and ref. number in []	Country of origin	Study type	Number of Included patients	Age of pts (years)	Females: males
Quality of life after COVID-19-induced critical illness: do the old survivors suffer more? Conings, 2021 [27]	Belgium	Retrospective observational mono-centric study	58	> 65	21:37 36%
Association between frailty and short- and long-term outcomes among critically ill patients. Bagshaw, 2014 [24]	Canada	Multicenter prospective cohort study.	138	69 ± 10	66:72 48% 21:37 36%
Quality of life and mortality in older adults with sepsis after one-year follow-up: A prospective cohort study demonstrating the significant impact of frailty Dong, 2023 [30]	China	Prospective cohort study, mono-centric	137 75 completed the survey	81.5(67–89.8.8)	24:52 32%
Recovery after critical illness in patients aged 80 years or older: a multi-centre prospective observational cohort study. Heyland, 2015 [25]	Canada	Multi-centre prospective observational cohort study	610 40 withdrawn consent or refused to respond. 63 missed to follow up on 507	> 80	272:338 45%
Long-term health-related quality of life and independence among older survivors of serious injury. Pollack, 2023 [31]	USA	Prospective observational cohort study Single urban county hospital-level 1	126	74 (69–83)	45:81 36%
Health-related quality of life in older patients surviving ICU treatment for COVID-19: results from an international observational study of patients older than 70 years. Soliman, 2022 [29]	International	Prospective observational study	707	74 (72–77)	220:487 31%
Functional Status and Quality of Life in Elderly Intensive Care Unit Survivors. Villa, 2016 [32]	Spain	Prospective 18-month observational study, mono-centre.	110 discharge from hospital 102 at 3-month follow-up 94 at 12-month follow-up	81 (78–84)	43:59 42%
Self-perceived recovery and quality of life in elderly patients surviving ICU admission for abdominal sepsis Cuijpers, 2022 [28]	The Netherlands	Cross-sectional retrospective survey on a prospectively recorded database Mono-centric	48	75(72–81)	13:35 27%
Critically ill octogenarians and nonagenarians: Evaluation of long-term outcomes, posthospital trajectories and quality of life one year and seven years after ICU discharge. Oeyen, 2017 [26]	Belgium	Prospective observational cohort analysis Multi-center	93 hospital survivors 7 died within 3 months after 6 died within 1 year after 45 died within 7 years after	83 (81–85)	36:57 39%

In all studies except one, the follow-up time was more than 12 months (Table 3).

**Table 3:** Data extraction characteristics of frailty, QoL and Health-related outcomes assessments

Follow-up time (Month) Ref. number in []	Frailty assessment tools.	Scales and Tools used to measure health-related outcomes – Quality of life. (Measurement methods)	Health-related outcomes parameters. (What are they looking at?)	Most affected impacted QoL domains
6 [27]	Rockwood Clinical Frailty Scale (CFS)	Telephone interview about self-reported HQoL Mortality EQ-5D-5 L questionnaire.	Post-traumatic stress disorder and general well-being All parameters used in EQ-5D-5 L	Decline in functional outcomes
12 [24]	Clinical Frailty Scale	Mortality, Short-Form Health Survey [SF-12], EuroQol Health Questionnaire [EQ-5D].	All-cause in-hospital mortality, death in ICU, hospital or at 6 months; health-related quality of life at 6 and 12 months.	All physical and mental health domains
12 [30]	Clinical Frailty Scale	Survival, EQ-5D, SF-12	Parameters used for EQ-5D and SF-12	Mobility problems
12 [25]	CGA questionnaire for Calculation of the frailty index	Telephone interviews of patients or relatives, SF-36	All parameters in SF-36	Not specified for individual domains
12 [31]	CFS (also proxies), CGA	EQ-5D-5 L ADL IADL Katz index ADL IADL Investigator-developed questions about post-injury recovery and complications (Have you returned to your pre-injury level of function? Were there any complications in your recovery process?)	Parameters used in EQ-5D-5 L, ADL, and IADL. Two investigator-developed questions about postinjury recovery and complications were included: “Have you returned to your preinjury level of function?” and “Were there any complications in your recovery process?” If a proxy respondent completed the survey, they were asked to answer the questions based on knowledge of the patient’s recovery process and current condition.	Activity limitations, persistent pain, and cognitive dysfunction.
12 [29]	CFS Katz’s activities of daily living	EurQol-5D-5 L, VAS	Parameters for EQ-5D-5 L The self-perceived quality of life. For study purposes, a level 4 answer or worse (‘I have severe/extreme problems’) as an outcome is considered unfavourable.	Not specified for individual domains
12 [32]	Barthel Index (BI)	SF 36 (Spanish version)	Parameters used in the SF-36 Spanish version	Physical functions
12–72 [28]	Modified Katz Index of Activities of Daily Living (Katz-ADL). Post-ICU frailty: Groningen Frailty Indicator (GFI)	Revised cardiac risk index (RCRI), Acute Physiology and Chronic Health Evaluation (APACHE) IV. The Dutch EQ-5D-3 L; EQ-VAS.	Mobility, self-care, usual activities, pain/discomfort and anxiety/depression.	Mobility and usual activities.
12 (12–84) [26]	ADL	SF-36 EuroQoL-5D (EQ-5D) After 7 years, patients were questioned about their social network, medical follow-up, financial situation, and happiness.	Parameters in SF-36: Questions concerning living situation, memories, sleep quality, and willingness to be readmitted to an ICU department. And medical follow-up after 7 years.	Physical functioning and self-care

### Frailty assessment tools

Among the various techniques used, Rockwood’s Clinical Frailty Score (CFS) was used in five studies [24, 27, 29–31]. One used the Modified Katz Index of Activities of Daily Living (Katz-ADL) [28], one study used Activity of Daily Living (ADL) as a proxy for frailty [26], one used the Post-ICU frailty: Groningen Frailty Indicator (GFI) [28], and the CGA questionnaire for calculating the frailty index [25] and Barthel Index (BI) [32]. While six studies used a single method to assess frailty, three employed two tools (Table 3, Additional file 2).

### Quality of life assessment tools

The tools used to measure QoL also showed variation. Approximately half of the studies used only scales [28–32], and the remaining used a combination of scales and other forms of questionnaires via telephone interviews or information letters [24–27].

The nine studies used different tools and scales to assess health-related outcomes and QoL. Only Villa [32] used one form — the SF-36. All others used a combination of either interviews or letters with questionnaires regarding residential status, functional status, and scales. Two studies used SF-12 and EQ-5D questionnaires [24, 30]. Conings supplied the EQ-5D questionnaire with a telephone interview about self-reported health-related quality of life (HRQoL) [27].

The EQ-5D was the most used tool, featured in six of the nine included studies. Three of the six used the EQ-5D-5 L [27, 29, 31], and one of the six used the EQ-5D-3 L [28] (Table 3, Additional file 3).

### Health-related parameters used in the assessment of QoL

All nine studies encompassed a range of parameters according to the standard forms in EQ-5D and SF-12/36, specified in Additional file 2. The studies that used or supplied interviews and telephone calls used more parameters - post-traumatic stress disorders, general well-being, and social [26, 27].

Bagshaw and Dong used the parameters in EQ-5D and SF-12 [24, 30]. Conings used the parameters in EQ-5D-5 L and the parameters in self-reported HRQoL [27]. Cuijpers used only EQ-5D [28],. Heyland and Villa used all parameters in SF-36 [25, 32]. Oeyen had the combination of all parameters in SF-36 and the question about willingness to be readmitted to the ICU department if it should be necessary again [26]. Pollack used all parameters in EQ-5D-5 L, ADL, and IADL (A part of ADL according to instrumental activities of daily living, such as shopping, cooking, and using public transportation) [31]. Two investigator-developed questions about post-injury recovery and complications, “Have you returned to your pre-injury level of function?” and “Were there any complications in your recovery process?” were used by Pollack et al. [31]. In the multinational study from Soliman, they used only parameters for EQ-5D-5 L. For study purposes, they considered a self-reported quality of life rating of level 4 or worse (“I have severe/extreme problems”) an unfavourable outcome [29] (Table 3).

### Patients’ perspectives on QoL post-ICU discharge

Pollack et al. found that some patients expressed dissatisfaction with their inability to engage in activities beyond basic daily tasks, which they had previously enjoyed before admission to the ICU [31].

Some patients reported a willingness to undergo readmission to the intensive care unit despite experiencing a deterioration in both their physical and mental health [26].

### Frailty and QoL

All included studies found that frailty is a strong predictor of poorer quality of life, increased disability, and higher mortality following critical illness. Frail patients are less likely to regain independence and are more susceptible to long-term physical and psychological challenges. Moreover, they tend to have lower functional status compared to non-frail patients.

## Discussion

Frailty and QoL are increasingly distinguished as crucial factors for ensuring accurate, fair, and adequate healthcare, facilitating early interventions, and improving patient outcomes [33–35]. Despite a comprehensive systematic literature search for this scoping review, the search identified only nine existing studies with assessment methods for both frailty and QoL in frail older patients following ICU discharge. This lack of research on both topics highlights the difficulty of accurately measuring frailty and QoL, primarily because these concepts are often related and only sometimes clearly defined in medical research.

The nine included studies came from six different countries, demonstrating that this topic has focus and interest worldwide.

The included studies report that various tools are used to assess frailty. Further, it is also established through the reviews by Dent et al. [36] and Bertschi et al. [5]. They noted that using a range of frailty measures makes it challenging to reach a consensus on the most appropriate tool for this population, and there is a need to harmonise the assessment methods. Standardisation is crucial for ensuring reliability, improvement in clinical outcomes, and fulfilling regulatory standards; however, scarce literature was found to support the arguments [33, 37].

The most recent review confirms that CFS is the most extensively and best-studied method for ICU patients [5]. It is easy and reliable to use, has substantial predictive value, has shown correlations to outcomes after discharge following critical illness, and can be combined with other methods [37, 38]. Some included studies used combined approaches, such as integrating the CFS with additional functional scales like Katz’s Activities of Daily Living (Katz-ADL), reflecting the multidimensional nature of frailty encompassing physical, cognitive, and social domains. This variation presents a challenge when comparing the methods used.

For QoL, there was also diversity in the tools used and scales applied. In this scoping review, we found that the most used form was EQ-5D, which was supplied with other instruments, including interviews or self-reported health status surveys. QoL is undoubtedly subjective [26]. This might explain why defining QoL only from descriptive or visual definitions can be difficult.

EQ-5D is a scale that assesses QoL using five dimensions with three to five levels. In the included studies, both 3 L and 5 L versions were used, even though the 5 L version apparently increases the precision of the assessment of QOL. In fact, EQ-5D-5 L is found to be superior to EQ-5D-3 L concerning various measurement properties, enabling improved sensitivity and accuracy in health status measurement [39]. It is not easy to consider evidence about the best assessment method for QoL, but EQ-5D is feasible due to its adequate psychometric properties [33].

Cuijpers’ research has demonstrated that although older patients frequently experience a decline in functional ability following ICU discharge, many still express willingness to undergo ICU treatment again [28]. While lower functional capacity is associated with reduced HRQoL scores, other studies of post-ICU survivors have demonstrated that standard HRQoL measures do not fully capture the aspects of life that patients themselves consider most important to their overall quality of life [31, 40–42]. This suggests that reduced functional capacity does not necessarily equate to a diminished quality of life, and that conventional HRQoL measures may be limited in their ability to adequately capture the broader dimensions of QoL adequately. These tools often fail to account for factors such as social context, individual expectations, and psychological adaptation [28].

As with frailty, various methodologies can cause variability in reported outcomes and make it difficult to reach a consensus across research studies.

In the present scoping review, most included studies indicated that increased frailty caused individuals to experience a lower QoL. This can be explained by the fact that increased frailty generally makes people more vulnerable and increases the risk of depression and a decline in physical functions [16, 43–46]. However, a rise in QoL has also been observed [26], which can also be driven by the fact that QoL can improve as included patients adapt to a new situation. Adjustment can lead to a better understanding of circumstances and the development of coping strategies. This might improve QoL in new contexts, which has been reported in patients with heart failure [47].

Furthermore, all the studies in our review showed a primary focus on health-related complications and resource utilisation. Very few have addressed the issue of patients’ perspectives and focused on using patient-related outcomes measurements, which can help identify what matters to patients and assess whether healthcare delivery meets those needs, encouraging a shift toward more meaningful metrics in clinical practice and healthcare systems [48].

Moreover, some studies also investigated the basis of patients’ perspectives towards QoL, such as patients’ spiritual or religious beliefs and practices, which can be essential to providing better QoL about life satisfaction [49, 50].

Although mortality is undoubtedly a major contributing factor, other potential reasons that can explain the substantial dropout from the included studies, including the participants’ inability to engage in follow-up assessment due to various reasons, are found [51]. These groups of patients often have disabilities, such as sensory impairments, which are also associated with poor health outcomes, including, among others, a higher risk of developing depressive symptoms, cognitive decline, and dementia [52, 53]. This may be a problem in telephone interviews or questionnaires, raising questions about the quality of their answers and making it difficult for them to participate in clinical trials. This could potentially lead to an overestimated QoL in the reported studies, as it raises the question of whether those who declined to respond were the ones experiencing the lowest QoL. They may have been less likely to complete follow-up assessments.

Numerous factors influence patients’ self-reporting regarding their current state and future expectations, mainly when multiple parameters are evaluated concurrently at a given moment [54]. This can be the same here for QoL because the parameters can be very subjective and qualitative and, therefore, difficult to assess [55].

The present scoping review only identified a limited number of studies examining frailty and QoL together, which is notable, given the strong association between these two factors. Frailty, typically linked to diminished physiological reserves and increased vulnerability to adverse outcomes, significantly affects QoL. However, the lack of combined assessment in current research represents a crucial gap in understanding the comprehensive experience of frail older ICU survivors. Future research could benefit from larger sample sizes. It should address this gap by developing integrated frameworks that simultaneously evaluate both frailty and QoL, as well as a broader approach focusing on other topics such as family relations, spirituality, and the ability to cope with frailty. This approach would offer a more complete understanding of the challenges of factors influencing QoL in this population and support more informed clinical decision-making.

### Strengths and limitations

One of the strengths of this scoping review was the systematic literature search using a broad search strategy in four databases. The search was repeated to ensure that articles were captured across different settings. A PRISMA_P flowchart was used to transparently describe the screening and exclusion process to ensure rigorous reproducibility.

The small number of eligible studies highlights the importance of more research in this area. The included studies were from six countries, showing the global interest in frailty and QoL for older people discharged from the ICU. Qualitative, quantitative, prospective, and retrospective studies were included to achieve a more nuanced and comprehensive understanding of the topics in different settings.

Several limitations deserve to be mentioned. Restrictions on language, publication period, and age can have limited the inclusion of some potentially relevant studies. Only nine eligible studies were found specific to the topic, limiting the findings’ generalizability. Moreover, the variety of assessment methods and tools used for frailty and QoL makes it challenging to compare and reach a consensus on the best practice.

Internationally, frailty is defined as a multidimensional condition involving both physical, social, functional and cognitive domains. Some tools identified in our review (e.g. Modified Katz ADL, and Barthel Index) primarily focus on functional ability and may not capture the full breadth of frailty. This limitation should be considered when interpreting our findings.

Even using a broad search string, some studies can be missed because QoL has many definitions, and many keywords can describe this topic. This scoping review focuses on frail older adults who can have difficulties in follow-up studies because of their health condition. This can cause a more considerable dropout among the frailest patients and, therefore, an overestimation of QoL. This might cause a bias in understanding the actual impact of frailty on QoL and vice versa. Although this scoping review included studies worldwide, it does not describe how cultural, regional, or healthcare system differences affect frailty or QoL. The lack of a unified framework for both topics makes it complex to interpret the results.

A limitation of our review is that we included all studies addressing frail patients, regardless of whether frailty was assessed before, during, or after ICU admission. While this approach ensured comprehensive inclusion, it may also have introduced heterogeneity across studies, which should be considered when interpreting the findings.

## Conclusion

Older, frail ICU survivors often experience persistent physical, cognitive, and pain-related changes in life that influence their QoL. However, current assessment tools insufficiently capture subjective dimensions, limiting the validity of reported QoL measurements. The heterogeneity of available instruments further complicates consensus and comparability across studies. Moreover, the limited number of studies examining both frailty and QoL makes it difficult to establish robust evidence for appropriate assessment methods. These findings underscore the complexity of the issue, and future research should therefore prioritise the development of standardised frameworks that integrate multidimensional and patient-reported perspectives to more accurately reflect what truly matters to patients in terms of QoL.

## Supplementary Information

Supplementary Material 1. Additional file 1: Search strings for Embase, MEDLINE, Psych-INFO, and CINAHL. Additional file 2: Frailty Assessment methods. Additional file 3: Quality of Life assessment methods.

Supplementary Material 2. PRISMA Flow Chart.

## Data Availability

No datasets were generated or analysed during the current study.

## Abbreviations

ICU: Intensive Care Unit
QoL: Quality of life
Embase: Excerpta Medica Database
MEDLINE: Medical Literature Analysis and Retrieval System Online
Psych-INFO: Psychological Information
CINAHL: Cumulative Index to Nursing and Allied Health Literature
PRISMA-P: Reporting Items for Systematic Review and Meta-AnalysisProtocols
SF-12: 12-Item Short Form Survey
SF-36: 36-Item Short Form Survey
BMJ: British Medical Journal
JBI: Joanna Briggs Institute
MESH: Medical Subject Headings
GPT: Generative Pre-trained Transformer
CFS: Clinical Frailty Score
GFI: Groningen Frailty Indicator
Katz-ADL: Katz Index of Activities of Daily Living
ADL: Activities of Daily Living
BI: Barthel Index
EQ-5D: EuroQol - 5 Domain
EQ-5D-3L: EuroQoL – 5 Domain – 3 Levels
EQ-5D-5L: EuroQol - 5 Domain – 5 Levels
HRQoL: Health-related quality of life
