# Supplementary material for: Frailty and quality of life in older ICU survivors: a scoping review of assessment tools and methodologies
Source: BMC Geriatr. 2026 Feb 10;26:616. doi: 10.1186/s12877-026-07104-7 (PMC13134141; doi:10.1186/s12877-026-07104-7)
Supplement: Supplementary file 1 — Supplementary Material 1. Additional file 1: Search strings for Embase, MEDLINE, Psych-INFO, and CINAHL. Additional file 2: Frailty Assessment methods. Additional file 3: Quality of Life assessment methods. [file 12877_2026_7104_MOESM1_ESM.pdf]

Supplementary Data

Additional file 1: Search strings

Ovid Embase

1	frailty/	34326
2	frail elderly/	13037
3	very elderly/	335822
4	geriatrics/	43590
5	geriatric assessment/	22950
6	aged/	4156873
7	older/	5
8	octogenarians/	335822
9	octogenarians.ti,ab,kf.	5610
10	nonagenarians/	321241
11	nonagenarian*.ti,ab,kf.	2836
12	older.ti,ab,kf.	874273
13	frail*.ti,ab,kf.	65447
14	elder*.ti,ab,kf.	484189
15	geriatric*.ti,ab,kf.	117791
16	old age*.ti,ab,kf.	54625
17	old* patient*.ti,ab,kf.	156223
18	senior*.ti,ab,kf.	81164
19	old* people.ti,ab,kf.	62755
20	((geriatric? or senior? or elderly or old*) adj2 (person? or people or adult? or patient?)).ti,ab,kf.	723492
21	(frail* adj2 (adult* or elder* or old or senior? or person? or people or patient?)).ti,ab,kf.	25206
22	exp shock/	197413
23	*multiple organ failure/	7216
24	exp sepsis/	381142
25	exp intensive care unit/	338108
26	exp critical illness/	38272
27	exp *intensive care/	309607
28	critical care unit*.ti,ab,kf.	7784
29	icu.ti,ab,kf.	188172
30	intensive therapy unit*.ti,ab,kf.	964
31	intensive treatment unit*.ti,ab,kf.	189
32	hospital emergency service*.ti,ab,kf.	775
33	intensive care*.ti,ab,kf.	319793
34	(critical* adj (care or ill*)).ti,ab,kf.	178794
35	multiple organ failure.ti,ab,kf.	12990
36	sepsis (ti,ab,kf)	213554
37	22 or 23 or 24 or 25 or 26 or 27 or 28 or 29 or 30 or 31 or 32 or 33 or 34 or 35 or 36	1265711
38	survivor/	74165
39	hospital discharge/	204044
40	survivor* (ti,ab,kf)	210522
41	hospital discharge (ti,ab,kf)	59863
42	patient discharge (ti,ab,kf)	4398
43	(after adj3 discharge).ti,ab,kf.	73517

44	((after or post or discharge or surviv* or follow* up) adj3 (critical* adj (care or ill*))).ti,ab,kf.	6446
45	(post adj3 discharge).ti,ab,kf.	23626
46	(following adj3 discharge).ti,ab,kf.	14074
47	((after or post or discharge or surviv* or follow* up) adj3 (intensive care or intensive treatment or icu)).ti,ab,kf.	30999
48	((after or post or discharge or surviv* or follow* up) adj3 (sepsis or septic shock)).ti,ab,kf.	16440
49	((after or post or discharge or surviv* or follow* up) adj3 (multiple organ*failure or multiple organ* dysfunction)).ti,ab,kf.	203
50	38 or 39 or 40 or 41 or 42 or 43 or 44 or 45 or 46 or 47 or 48 or 49	523677
51	exp quality of life/	729510
52	activities of daily living/	105039
53	daily live activity/	0
54	daily live activity (ti,ab,kf)	4
55	quality adjusted life year/	38853
56	quality adjusted life year (ti,ab,kf)	10194
57	personal autonomy/	15832
58	personal autonomy (ti,ab,kf)	1115
59	happiness/	13664
60	happiness (ti,ab,kf)	12782
61	self-concept/	119769
62	self-concept (ti,ab,kf)	7538
63	family-relation (ti,ab,kf)	134
64	family-relation/	13858
65	religion/	83388
66	religion (ti,ab,kf)	22954
67	social-support/	126020
68	social-support (ti,ab,kf)	73783
69	activities of daily living (ti,ab,kf)	54763
70	quality of life (ti,ab,kf)	672608
71	life quality (ti,ab,kf)	20689
72	satisfaction/	85198
73	satisfaction (ti,ab,kf)	281876
74	life satisfaction/	13806
75	life satisfaction (ti,ab,kf)	14108
76	patient satisfaction/	183564
77	patient satisfaction (ti,ab,kf)	77499
78	patient preference/	29610
79	patient preference (ti,ab,kf)	11075
80	Financial Support/	121422
81	Financial Support (ti,ab,kf)	12363
82	coping/	3534
83	coping (ti,ab,kf)	101601
84	positive experience (ti,ab,kf)	4392
85	fear of death (ti,ab,kf)	1753
86	personal satisfaction (ti,ab,kf)	1342
87	patient experience (ti,ab,kf)	20466
88	51 or 52 or 53 or 54 or 55 or 56 or 57 or 58 or 59 or 60 or 61 or 62 or 63 or 64 or 65 or 66 or 67 or 68 or 69 or 70 or 72 or 73 or 74 or 75 or 76 or 77 or 78 or 79 or 80 or 81 or 82 or 83 or 84 or 85 or 86 or 87	1827308
89	1 or 2 or 3 or 4 or 5 or 6 or 7 or 8 or 9 or 10 or 11 or 12 or 13 or 14 or 15 or 16 or 17 or 18 or 19 or 20 or 21	4983241
90	37 and 50 and 88 and 89	2851
91	limit 90 to yr="1947 - 2023"	2568

Ovid Medline

1	Frailty/	12076
2	Frail Elderly/	16641
3	Aged/	3550559
4	"Aged, 80 and over"/	1051499
5	Geriatrics/	31849
6	Geriatric Assessment/	34096
7	nonagenarians/	152
8	nonagenarian*.ti,ab,kf.	1929
9	octogenarians.ti,ab,kf.	3345
10	frail* (ti,ab,kf)	42677
11	elder* (ti,ab,kf)	330774
12	geriatric* (ti,ab,kf)	84272
13	old age* (ti,ab,kf)	37446
14	old* adult (ti,ab,kf)	16129
15	old* patient* (ti,ab,kf)	99824
16	senior* (ti,ab,kf)	56630
17	old* people (ti,ab,kf)	48461
18	((geriatric? or senior? or elderly or old*) adj2 (person? or people or adult? or patient?)) (ti,ab,kf)	502987
19	(frail* adj2 (adult* or elder* or old or senior? or person? or people or patient?)) (ti,ab,kf)	15818
20	1 or 2 or 3 or 4 or 5 or 6 or 7 or 8 or 9 or 10 or 11 or 12 or 13 or 14 or 15 or 16 or 17 or 18 or 19	3943543
21	exp Shock/	89108
22	*Multiple Organ Failure/	6131
23	exp Sepsis/	148507
24	exp Intensive Care Units/	113208
25	exp Critical Illness/	41827
26	exp Critical Care/	69117
27	critical care unit* (ti,ab,kf)	4496
28	intensive therapy unit* (ti,ab,kf)	693
29	intensive treatment unit* (ti,ab,kf)	88
30	icu (ti,ab,kf)	95818
31	hospital emergency service* (ti,ab,kf)	815
32	intensive care* (ti,ab,kf)	215343
33	(critical* adj (care or ill*)) (ti,ab,kf)	113363
34	sepsis (ti,ab,kf)	132232
35	multiple organ failure (ti,ab,kf)	8750
36	21 or 22 or 23 or 24 or 25 or 26 or 27 or 28 or 30 or 31 or 32 or 34 or 35	574514
37	Survivors/	32123
38	Patient Discharge/	42488
39	hospital discharge (ti,ab,kf)	37714
40	patient discharge (ti,ab,kf)	2764
41	((after or post or discharge or surviv* or follow* up) adj3 (critical* adj (care or ill*))) (ti,ab,kf)	3971
42	((after or post or discharge or surviv* or follow* up) adj3 (intensive care or intensive treatment or icu)) (ti,ab,kf)	17214
43	((after or post or discharge or surviv* or follow* up) adj3 (sepsis or septic shock)) (ti,ab,kf)	9677
44	((after or post or discharge or surviv* or follow* up) adj3 (multiple organ*failure or multiple organ* dysfunction)) (ti,ab,kf)	172
45	Survivor* (ti,ab,kf)	143068
46	(after adj3 discharge).ti,ab,kf.	44502
47	(post adj3 discharge) (ti,ab,kf)	11477
48	(following adj3 discharge).ti,ab,kf.	8009
49	37 or 38 or 39 or 40 or 41 or 42 or 43 or 44 or 45 or 46 or 47 or 48	275308
50	exp "Quality of Life"/	297337
51	Life quality (ti,ab,kf)	11939
52	"Activities of Daily Living"/	75802
53	Activities of Daily living (ti,ab,kf)	39025
54	daily live activity (ti,ab,kf)	1
55	quality of life (ti,ab,kf)	431908

56	patient satisfaction/	92810
57	patient satisfaction (ti,ab,kf)	53047
58	Quality-Adjusted Life Years/	17094
59	Quality-Adjusted Life Years (ti,ab,kf)	12535
60	Personal autonomy/	18828
61	Personal autonomy (ti,ab,kf)	881
62	Happiness/	5900
63	Happiness (ti,ab,kf)	10772
64	Patient preference (ti,ab,kf)	6506
65	fear of death (ti,ab,kf)	1196
66	self-concept/	62359
67	self-concept (ti,ab,kf)	6649
68	Family Relations/	12243
69	Family Relations (ti,ab,kf)	1567
70	religion/	16211
71	Religion (ti,ab,kf)	20378
72	social support/	82214
73	social support (ti,ab,kf)	60629
74	financial support/	3954
75	financial support (ti,ab,kf)	6890
76	positive experience (ti,ab,kf)	2891
77	coping (ti,ab,kf)	79716
78	Satisfaction (ti,ab,kf)	203884
79	life satisfaction (ti,ab,kf)	12594
80	personal satisfaction (ti,ab,kf)	964
81	patient experience (ti,ab,kf)	11848
82	50 or 51 or 52 or 53 or 54 or 55 or 56 or 57 or 58 or 59 or 60 or 61 or 62 or 63 or 64 or 65 or 66 or 67 or 68 or 69 or 70 or 71 or 72 or 73 or 74 or 75 or 76 or 77 or 78 or 79 or 80 or 81	1078226
83	20 and 36 and 49 and 82	1395
84	limit 83 to yr="2023"	42

APA PsycInfo

1	Aging/	74696
2	Geriatrics/	13406
3	Geriatric Assessment/	1238
4	"Aged (Attitudes Toward)"/	1939
5	Geriatric Patients/	14559
6	Health Impairments/	4398
7	frail* (ti,ab,id)	6614
8	Elder* (ti,ab,id)	80771
9	geriatric* (ti,ab,id)	21051
10	old age* (ti,ab,id)	16530
11	old* adult (ti,ab,id)	8154
12	old* patient* (ti,ab,id)	14160
13	senior* (ti,ab,id)	33519
14	old* people (ti,ab,id)	19904
15	((geriatric? or senior? or elderly or old*) adj2 (person? or people or adult? or patient?)) (ti,ab,id)	142367
16	(frail* adj2 (adult* or elder* or old or senior? or person? or people or patient?)) (ti,ab,id)	2754
17	age* (ti,ab,id)	1096331
18	1 or 2 or 3 or 4 or 5 or 6 or 7 or 8 or 9 or 10 or 11 or 12 or 13 or 14 or 15 or 16 or 17	1209541
19	exp Shock/	4328
20	exp Sepsis/	332
21	exp *Intensive Care/	6602
22	Critical Illness/	615
23	shock (ti,ab,id)	23594
24	sepsis (ti,ab,id)	1225
25	intensive care* (ti,ab,id)	11525
26	critical care unit* (ti,ab,id)	404
27	critical care* (ti,ab,id)	2896
28	multiple organ failure (ti,ab,id)	53
29	(critical* adj (care or ill*)) (ti,ab,id)	4677
30	hospital emergency service* (ti,ab,id)	75
31	icu (ti,ab,id)	3661
32	intensive therapy unit* (ti,ab,id)	19
33	intensive treatment unit* (ti,ab,id)	18
34	19 or 20 or 21 or 22 or 23 or 24 or 25 or 26 or 27 or 28 or 29 or 30 or 31 or 32 or 33	40454
35	survivors/	19600
36	Hospital Discharge/	3479
37	Survivor* (ti,ab,id)	40902
38	patient discharge (ti,ab,id)	403
39	hospital discharge (ti,ab,id)	4414
40	((after or post or discharge or surviv* or follow* up) adj3 (critical* adj (care or ill*))) (ti,ab,id)	273
41	((after or post or discharge or surviv* or follow* up) adj3 (intensive care or intensive treatment or icu)) (ti,ab,id)	1018
42	((after or post or discharge or surviv* or follow* up) adj3 (sepsis or septic shock)) (ti,ab,id)	154
43	((after or post or discharge or surviv* or follow* up) adj3 (multiple organ*failure or multiple organ* dysfunction)) (ti,ab,id)	1
44	(following adj3 discharge) (ti,ab,id)	1910
45	35 or 36 or 37 or 38 or 39 or 40 or 41 or 42 or 43 or 44	50305
46	exp "Quality of Life"/	56888
47	exp "Activities of Daily Living"/	7334

48	Activities of Daily living (ti,ab,id)	12397
49	personal satisfaction (ti,ab,id)	647
50	quality of life (ti,ab,id)	95269
51	patient satisfaction/	6916
52	patient satisfaction (ti,ab,id)	5930
53	coping (ti,ab,id)	101016
54	Quality-Adjusted Life Years (ti,ab,id)	1297
55	Personal autonomy (ti,ab,id)	820
56	happiness/	10570
57	happiness (ti,ab,id)	20218
58	self-concept/	47008
59	self-concept (ti,ab,id)	26257
60	fear of death (ti,ab,id)	1805
61	family relation/	40776
62	family relation (ti,ab,id)	113
63	Religion/	22288
64	Religion (ti,ab,id)	40147
65	social support/	47220
66	social support (ti,ab,id)	64338
67	Satisfaction/	14688
68	Satisfaction (ti,ab,id)	138506
69	life satisfaction/	13856
70	life satisfaction (ti,ab,id)	19459
71	patient preference (ti,ab,id)	862
72	Financial Support (ti,ab,id)	2939
73	Life quality (ti,ab,id)	2036
74	positive experience (ti,ab,id)	2064
75	patient experience (ti,ab,id)	2152
76	46 or 47 or 48 or 49 or 50 or 51 or 52 or 53 or 54 or 55 or 56 or 57 or 58 or 59 or 60 or 61 or 62 or 63 or 64 or 65 or 66 or 67 or 68 or 69 or 70 or 71 or 72 or 73 or 74 or 75	537672
77	18 and 34 and 45 and 76	137
78	limit 77 to yr="2023"	10

EBSCO Cinahl

EBSCOhost

#	Query	Limiters/Expanders	Last Run Via	Results
S88	S22 AND S52 AND S53 AND S87	Expanders - Apply equivalent subjects<br>Search modes - Proximity	Interface - EBSCOhost Research Databases<br>Search Screen - Advanced Search<br>Database - CINAHL with Full Text	645
S87	S54 OR S55 OR S56 OR S57 OR S58 OR S59 OR S60 OR S61 OR S62 OR S63 OR S64 OR S65 OR S66 OR S67 OR S68 OR S69 OR S70 OR S71 OR S72 OR S73 OR S74 OR S75 OR S76 OR S77 OR S78 OR S79 OR S80 OR S81 OR S82 OR S83 OR S84 OR S85 OR S86	Expanders - Apply equivalent subjects<br>Search modes - Proximity	Interface - EBSCOhost Research Databases<br>Search Screen - Advanced Search<br>Database - CINAHL with Full Text	Display
S86	TI life quality OR AB life quality	Expanders - Apply equivalent subjects<br>Search modes - Proximity	Interface - EBSCOhost Research Databases<br>Search Screen - Advanced Search<br>Database - CINAHL with Full Text	Display
S85	TI fear of death OR AB fear of death	Expanders - Apply equivalent subjects<br>Search modes - Proximity	Interface - EBSCOhost Research Databases<br>Search Screen - Advanced Search<br>Database - CINAHL with Full Text	Display
S84	TI personal satisfaction OR AB personal satisfaction	Expanders - Apply equivalent subjects<br>Search modes - Proximity	Interface - EBSCOhost Research Databases<br>Search Screen - Advanced Search<br>Database - CINAHL with Full Text	Display
S83	TI Financial Support OR AB Financial Support	Expanders - Apply equivalent subjects<br>Search modes - Proximity	Interface - EBSCOhost Research Databases<br>Search Screen - Advanced Search	Display
S82	TI positive experience OR positive experience	Expanders - Apply equivalent subjects<br>Search modes - Proximity	Interface - EBSCOhost Research Databases<br>Search Screen - Advanced Search<br>Database - CINAHL with Full Text	Display
S81	TI patient preference OR AB patient preference	Expanders - Apply equivalent subjects<br>Search modes - Proximity	Interface - EBSCOhost Research Databases<br>Search Screen - Advanced Search<br>Database - CINAHL with Full Text	Display
S80	TI life satisfaction OR AB life satisfaction	Expanders - Apply equivalent subjects<br>Search modes - Proximity	Interface - EBSCOhost Research Databases<br>Search Screen - Advanced Search<br>Database - CINAHL with Full Text	Display
S79	TI Satisfaction OR AB Satisfaction	Expanders - Apply equivalent subjects<br>Search modes - Proximity	Interface - EBSCOhost Research Databases<br>Search Screen - Advanced Search<br>Database - CINAHL with Full Text	Display
S78	TI social support OR AB social support	Expanders - Apply equivalent subjects<br>Search modes - Proximity	Interface - EBSCOhost Research Databases<br>Search Screen - Advanced Search<br>Database - CINAHL with Full Text	Display
S77	TI religion OR AB religion	Expanders - Apply equivalent subjects<br>Search modes - Proximity	Interface - EBSCOhost Research Databases<br>Search Screen - Advanced Search<br>Database - CINAHL with Full Text	Display
S76	TI family relation OR AB family relation	Expanders - Apply equivalent subjects<br>Search modes - Proximity	Interface - EBSCOhost Research Databases<br>Search Screen - Advanced Search<br>Database - CINAHL with Full Text	Display
S75	TI self-concept OR AB self-concept	Expanders - Apply equivalent subjects<br>Search modes - Proximity	Interface - EBSCOhost Research Databases<br>Search Screen - Advanced Search<br>Database - CINAHL with Full Text	Display
S74	TI happiness OR AB happiness	Expanders - Apply equivalent subjects<br>Search modes - Proximity	Interface - EBSCOhost Research Databases<br>Search Screen - Advanced Search	Display

S73	TI personal autonomy OR AB personal autonomy	Expanders - Apply equivalent subjects<br>Search modes - Proximity	Interface - EBSCOhost Research Databases<br>Search Screen - Advanced Search<br>Database - CINAHL with Full Text	Display
S72	TI quality adjusted life year OR AB quality adjusted life year	Expanders - Apply equivalent subjects<br>Search modes - Proximity	Interface - EBSCOhost Research Databases<br>Search Screen - Advanced Search<br>Database - CINAHL with Full Text	Display
S71	TI daily live activity OR AB daily live activity	Expanders - Apply equivalent subjects<br>Search modes - Proximity	Interface - EBSCOhost Research Databases<br>Search Screen - Advanced Search<br>Database - CINAHL with Full Text	Display
S70	TI coping OR AB coping	Expanders - Apply equivalent subjects<br>Search modes - Proximity	Interface - EBSCOhost Research Databases<br>Search Screen - Advanced Search<br>Database - CINAHL with Full Text	Display
S69	TI patient satisfaction OR AB patient satisfaction	Expanders - Apply equivalent subjects<br>Search modes - Proximity	Interface - EBSCOhost Research Databases<br>Search Screen - Advanced Search<br>Database - CINAHL with Full Text	Display
S68	patient experience	Expanders - Apply equivalent subjects<br>Search modes - Proximity	Interface - EBSCOhost Research Databases<br>Search Screen - Advanced Search<br>Database - CINAHL with Full Text	Display
S67	ti activities of daily living or ab activities of daily living	Expanders - Apply equivalent subjects<br>Search modes - Proximity	Interface - EBSCOhost Research Databases<br>Search Screen - Advanced Search<br>Database - CINAHL with Full Text	Display
S66	ti quality of life or ab quality of life	Expanders - Apply equivalent subjects<br>Search modes - Proximity	Interface - EBSCOhost Research Databases<br>Search Screen - Advanced Search<br>Database - CINAHL with Full Text	Display
S65	(MH "Financial Support")	Expanders - Apply equivalent subjects<br>Search modes - Proximity	Interface - EBSCOhost Research Databases<br>Search Screen - Advanced Search	Display
S55	(MH "Activities of Daily Living")	Expanders - Apply equivalent subjects<br>Search modes - Proximity	Interface - EBSCOhost Research Databases<br>Search Screen - Advanced Search<br>Database - CINAHL with Full Text	Display
S54	(MH "Quality of Life")	Expanders - Apply equivalent subjects<br>Search modes - Proximity	Interface - EBSCOhost Research Databases<br>Search Screen - Advanced Search<br>Database - CINAHL with Full Text	Display
S53	S38 OR S39 OR S40 OR S41 OR S42 OR S43 OR S44 OR S45 OR S46 OR S47 OR S48 OR S49 OR S50 OR S51	Expanders - Apply equivalent subjects<br>Search modes - Proximity	Interface - EBSCOhost Research Databases<br>Search Screen - Advanced Search<br>Database - CINAHL with Full Text	Display
S52	S23 OR S24 OR S25 OR S26 OR S27 OR S28 OR S29 OR S30 OR S31 OR S32 OR S33 OR S34 OR S35 OR S36 OR S37	Expanders - Apply equivalent subjects<br>Search modes - Proximity	Interface - EBSCOhost Research Databases<br>Search Screen - Advanced Search<br>Database - CINAHL with Full Text	Display
S51	TI survivor OR AB survivor	Expanders - Apply equivalent subjects<br>Search modes - Proximity	Interface - EBSCOhost Research Databases<br>Search Screen - Advanced Search<br>Database - CINAHL with Full Text	Display
S50	hospital discharge OR AB hospital discharge	Expanders - Apply equivalent subjects<br>Search modes - Proximity	Interface - EBSCOhost Research Databases<br>Search Screen - Advanced Search<br>Database - CINAHL with Full Text	Display
S49	TI survivorship OR AB survivorship	Expanders - Apply equivalent subjects<br>Search modes - Proximity	Interface - EBSCOhost Research Databases<br>Search Screen - Advanced Search<br>Database - CINAHL with Full Text	Display
S48	(TI post N3 discharge) OR (AB post N3 discharge)	Expanders - Apply equivalent subjects<br>Search modes - Proximity	Interface - EBSCOhost Research Databases<br>Search Screen - Advanced Search<br>Database - CINAHL with Full Text	Display
S47	(TI after N3 discharge) OR (AB after N3 discharge)	Expanders - Apply equivalent subjects<br>[illegible]	Interface - EBSCOhost Research Databases<br>[illegible]	Display

S46	(TI following N3 discharge) OR (AB following N3 discharge)	Expanders - Apply equivalent subjects<br>Search modes - Proximity	Interface - EBSCOhost Research Databases<br>Search Screen - Advanced Search<br>Database - CINAHL with Full Text	Display
S45	((after or post or discharge or surviv* or follow* up)N3 multiple organ* failure or multiple organ* dysfunction))	Expanders - Apply equivalent subjects<br>Search modes - Proximity	Interface - EBSCOhost Research Databases<br>Search Screen - Advanced Search<br>Database - CINAHL with Full Text	Display
S44	((after or post or discharge or surviv* or follow* up)N3 (sepsis or septic shock))	Expanders - Apply equivalent subjects<br>Search modes - Proximity	Interface - EBSCOhost Research Databases<br>Search Screen - Advanced Search<br>Database - CINAHL with Full Text	Display
S43	((after or post or discharge or surviv* or follow* up)N3(intensive care or intensive treatment or icu))	Expanders - Apply equivalent subjects<br>Search modes - Proximity	Interface - EBSCOhost Research Databases<br>Search Screen - Advanced Search<br>Database - CINAHL with Full Text	Display
S42	((after or post or cischarge or surviv* or follow* up)N3(critical* N1(care or ill*)))	Expanders - Apply equivalent subjects<br>Search modes - Proximity	Interface - EBSCOhost Research Databases<br>Search Screen - Advanced Search<br>Database - CINAHL with Full Text	Display
S41	TI patient discharge OR AB patient discharge	Expanders - Apply equivalent subjects<br>Search modes - Proximity	Interface - EBSCOhost Research Databases<br>Search Screen - Advanced Search<br>Database - CINAHL with Full Text	Display
S40	(MH "Survivorship")	Expanders - Apply equivalent subjects<br>Search modes - Proximity	Interface - EBSCOhost Research Databases<br>Search Screen - Advanced Search<br>Database - CINAHL with Full Text	Display
S39	(MH "Patient Discharge")	Expanders - Apply equivalent subjects<br>Search modes - Proximity	Interface - EBSCOhost Research Databases<br>Search Screen - Advanced Search<br>Database - CINAHL with Full Text	Display
S38	(MH "Survivors")	Expanders - Apply equivalent subjects<br>Search modes - Proximity	Interface - EBSCOhost Research Databases<br>Search Screen - Advanced Search	Display
S37	TI (multiple organ failure) OR AB (multiple organ failure)	Expanders - Apply equivalent subjects<br>Search modes - Proximity	Interface - EBSCOhost Research Databases<br>Search Screen - Advanced Search<br>Database - CINAHL with Full Text	Display
S36	TI sepsis OR AB sepsis	Expanders - Apply equivalent subjects<br>Search modes - Proximity	Interface - EBSCOhost Research Databases<br>Search Screen - Advanced Search<br>Database - CINAHL with Full Text	Display
S35	critical* N2" (care or ill*)	Expanders - Apply equivalent subjects<br>Search modes - Proximity	Interface - EBSCOhost Research Databases<br>Search Screen - Advanced Search<br>Database - CINAHL with Full Text	Display
S34	ti critical care unit* or ab critical care unit*	Expanders - Apply equivalent subjects<br>Search modes - Proximity	Interface - EBSCOhost Research Databases<br>Search Screen - Advanced Search<br>Database - CINAHL with Full Text	Display
S33	ti intensive therapy unit* or ab intensive therapy unit*	Expanders - Apply equivalent subjects<br>Search modes - Proximity	Interface - EBSCOhost Research Databases<br>Search Screen - Advanced Search<br>Database - CINAHL with Full Text	Display
S32	ti icu or ab icu	Expanders - Apply equivalent subjects<br>Search modes - Proximity	Interface - EBSCOhost Research Databases<br>Search Screen - Advanced Search<br>Database - CINAHL with Full Text	Display
S31	ti intensive treatment unit* or ab intensive treatment unit*	Expanders - Apply equivalent subjects<br>Search modes - Proximity	Interface - EBSCOhost Research Databases<br>Search Screen - Advanced Search<br>Database - CINAHL with Full Text	Display
S30	ti hospital emergency service or ab hospital emergency service	Expanders - Apply equivalent subjects<br>Search modes - Proximity	Interface - EBSCOhost Research Databases<br>Search Screen - Advanced Search<br>Database - CINAHL with Full Text	Display

S29	ti intensive care* or ab intensive care*	Expanders - Apply equivalent subjects<br>Search modes - Proximity	Interface - EBSCOhost Research Databases<br>Search Screen - Advanced Search<br>Database - CINAHL with Full Text	Display
S28	(MH "Critical Care")	Expanders - Apply equivalent subjects<br>Search modes - Proximity	Interface - EBSCOhost Research Databases<br>Search Screen - Advanced Search<br>Database - CINAHL with Full Text	Display
S27	(MH "Critical Illness")	Expanders - Apply equivalent subjects<br>Search modes - Proximity	Interface - EBSCOhost Research Databases<br>Search Screen - Advanced Search<br>Database - CINAHL with Full Text	Display
S26	(MH "Intensive Care Units")	Expanders - Apply equivalent subjects<br>Search modes - Proximity	Interface - EBSCOhost Research Databases<br>Search Screen - Advanced Search<br>Database - CINAHL with Full Text	Display
S25	(MH "Sepsis")	Expanders - Apply equivalent subjects<br>Search modes - Proximity	Interface - EBSCOhost Research Databases<br>Search Screen - Advanced Search<br>Database - CINAHL with Full Text	Display
S24	(MH "Multiple Organ Dysfunction Syndrome")	Expanders - Apply equivalent subjects<br>Search modes - Proximity	Interface - EBSCOhost Research Databases<br>Search Screen - Advanced Search<br>Database - CINAHL with Full Text	Display
S23	(MH "Shock+")	Expanders - Apply equivalent subjects<br>Search modes - Proximity	Interface - EBSCOhost Research Databases<br>Search Screen - Advanced Search<br>Database - CINAHL with Full Text	Display
S22	S1 OR S2 OR S3 OR S4 OR S5 OR S6 OR S7 OR S8 OR S9 OR S10 OR S11 OR S12 OR S13 OR S14 OR S15 OR S16 OR S17 OR S18 OR S19 OR S20 OR S21	Expanders - Apply equivalent subjects<br>Search modes - Proximity	Interface - EBSCOhost Research Databases<br>Search Screen - Advanced Search<br>Database - CINAHL with Full Text	Display
S21	TI nonagenarian* OR AB nonagenarian*	Expanders - Apply equivalent subjects<br>Search modes - Proximity	Interface - EBSCOhost Research Databases<br>Search Screen - Advanced Search<br>Database - CINAHL with Full Text	Display
S20	(MH "Nonagenarian*")	Expanders - Apply equivalent subjects<br>Search modes - Proximity	Interface - EBSCOhost Research Databases<br>Search Screen - Advanced Search<br>Database - CINAHL with Full Text	Display
S19	TI octogenarians OR AB octogenarians	Expanders - Apply equivalent subjects<br>Search modes - Proximity	Interface - EBSCOhost Research Databases<br>Search Screen - Advanced Search<br>Database - CINAHL with Full Text	Display
S18	(MH "Octogenarians")	Expanders - Apply equivalent subjects<br>Search modes - Proximity	Interface - EBSCOhost Research Databases<br>Search Screen - Advanced Search<br>Database - CINAHL with Full Text	Display
S17	TI (((geriatric? or senior? or elderly or old*) N2 (person? or people or adult? or patient?))) OR AB (((geriatric? or senior? or elderly or old*) N2 (person? or people or adult? or patient?)))	Expanders - Apply equivalent subjects<br>Search modes - Proximity	Interface - EBSCOhost Research Databases<br>Search Screen - Advanced Search<br>Database - CINAHL with Full Text	Display
S16	TI ((frail* N2 (adult* or elder* or old or senior? or person? or people or patient?))) OR AB ((frail* N2 (adult* or elder* or old or senior? or person? or people or patient?)))	Expanders - Apply equivalent subjects<br>Search modes - Proximity	Interface - EBSCOhost Research Databases<br>Search Screen - Advanced Search<br>Database - CINAHL with Full Text	Display
S15	TI old* people OR AB old* people	Expanders - Apply equivalent subjects<br>Search modes - Proximity	Interface - EBSCOhost Research Databases<br>Search Screen - Advanced Search<br>Database - CINAHL with Full Text	Display
S14	TI senior* OR AB senior*	Expanders - Apply equivalent subjects<br>Search modes - Proximity	Interface - EBSCOhost Research Databases<br>Search Screen - Advanced Search<br>Database - CINAHL with Full Text	Display

S13	TI old* patient* OR AB old* patient*	Expanders - Apply equivalent subjects<br>Search modes - Proximity	Interface - EBSCOhost Research Databases<br>Search Screen - Advanced Search<br>Database - CINAHL with Full Text	Display
S12	TI old* adult OR AB old* adult	Expanders - Apply equivalent subjects<br>Search modes - Proximity	Interface - EBSCOhost Research Databases<br>Search Screen - Advanced Search<br>Database - CINAHL with Full Text	Display
S11	TI old age* OR AB old age*	Expanders - Apply equivalent subjects<br>Search modes - Proximity	Interface - EBSCOhost Research Databases<br>Search Screen - Advanced Search<br>Database - CINAHL with Full Text	Display
S10	TI geriatric* OR AB geriatric*	Expanders - Apply equivalent subjects<br>Search modes - Proximity	Interface - EBSCOhost Research Databases<br>Search Screen - Advanced Search<br>Database - CINAHL with Full Text	Display
S9	TI elder* OR AB elder*	Expanders - Apply equivalent subjects<br>Search modes - Proximity	Interface - EBSCOhost Research Databases<br>Search Screen - Advanced Search<br>Database - CINAHL with Full Text	Display
S8	TI frail* OR AB frail*	Expanders - Apply equivalent subjects<br>Search modes - Proximity	Interface - EBSCOhost Research Databases<br>Search Screen - Advanced Search<br>Database - CINAHL with Full Text	Display
S7	(MH "Geriatric Functional Assessment")	Expanders - Apply equivalent subjects<br>Search modes - Proximity	Interface - EBSCOhost Research Databases<br>Search Screen - Advanced Search<br>Database - CINAHL with Full Text	Display
S6	(MH "Geriatric Assessment")	Expanders - Apply equivalent subjects<br>Search modes - Proximity	Interface - EBSCOhost Research Databases<br>Search Screen - Advanced Search<br>Database - CINAHL with Full Text	Display
S6	(MH "Geriatric Assessment")	Expanders - Apply equivalent subjects<br>Search modes - Proximity	Interface - EBSCOhost Research Databases<br>Search Screen - Advanced Search<br>Database - CINAHL with Full Text	Display
S5	(MH "Geriatrics")	Expanders - Apply equivalent subjects<br>Search modes - Proximity	Interface - EBSCOhost Research Databases<br>Search Screen - Advanced Search<br>Database - CINAHL with Full Text	Display
S4	(MH "Aged, 80 and Over")	Expanders - Apply equivalent subjects<br>Search modes - Proximity	Interface - EBSCOhost Research Databases<br>Search Screen - Advanced Search<br>Database - CINAHL with Full Text	Display
S3	(MH "Aged")	Expanders - Apply equivalent subjects<br>Search modes - Proximity	Interface - EBSCOhost Research Databases<br>Search Screen - Advanced Search<br>Database - CINAHL with Full Text	Display
S2	(MH "Frail Elderly")	Expanders - Apply equivalent subjects<br>Search modes - Proximity	Interface - EBSCOhost Research Databases<br>Search Screen - Advanced Search<br>Database - CINAHL with Full Text	Display
S1	(MH "Frailty Syndrome")	Expanders - Apply equivalent subjects<br>Search modes - Proximity	Interface - EBSCOhost Research Databases<br>Search Screen - Advanced Search<br>Database - CINAHL with Full Text	Display

Additional filed 2 Frailty assessment

Rockwood Clinical Frailty Scale

Rockwood Clinical Frailty scale is a tool developed by Dr. Kennth Rocwood for the Canadian Study of Health and Aging in 2005 used for assessing the level of elderlys frailty [1].

It is a clinical administered judgment-based scale, which means it relies on the professional evaluation of the patient. It evaluates specific domains including comorbidity, function and cognition.

It consists of 9 levels ranging from 1 to 9 – Very fit to terminally ill.

1. **Very Fit**
   Robust, active, energetic, and motivated people commonly exercise regularly. They are among the fittest for their age.
2. **Well**
   People with no active disease symptoms but less fit than category 1. Often, they exercise or are very active occasionally, e.g. seasonally.
3. **Managing Well**
   People whose medical problems are well controlled but are not regularly active beyond routine walking.
4. **Vulnerable**
   While not dependent on others for daily help, symptoms often limit activities. A common complaint is being “slowed up” and/or being tired during the day.
5. **Mildly Frail**
   These people often have more evident slowing and need help in high-order IADLs (finances, transportation, heavy housework, medications). Typically, mild frailty progressively impairs shopping and walking outside alone, meal preparation and housework.
6. **Moderately Frail**
   People need help with all outside activities and with keeping house. Inside, they often have problems with stairs, need help with bathing, and might require minimal assistance (curing, standby) with dressing.
7. **Severely Frail**
   Completely dependent on personal care, from whatever cause (physical or cognitive). Even so, they seem stable and not at high risk of dying (within ~ 6 months).
8. **Very Severely Frail**
   Completely dependent, approaching the end of life. Typically, they could not recover even from a minor illness.
9. **Terminally Ill**
   Approaching the end of life. This category applies to people with a life expectancy.

Karnofsky Performance Status

The Karnofsky Performance Status Scale (KPSS) was originally developed in 1948 to assess patients' ability to perform daily activities and their overall functional status. The scale ranges from 100% (No evidence of disease and able to carry out all normal activities) to 0% (death).

This evaluation relies primarily on physician input with little to no input from the patient. [2]

Functional Evaluation	Number	Karnofsky Scale
Functionally independent	100	Normal, no complaints, no evidence of disease
Able to carry on normal activity and to work; no special care needed	90	Able to carry on normal activity; minor signs or symptoms of disease
Frail	70	Cares for self; unable to carry on normal activity or to do active work
Unable to work; able to live at home and care for most personal needs; varying amount of assistance needed. Limited treatment	60	Requires occasional assistance but is able to care for most of his personal needs
Basic restorative as well as maintenance and monitoring	50	Requires considerable assistance and frequent medical care
Functionally dependent	40	Disabled; requires special care and assistance.
Unable to care for self; requires equivalent of institutional or hospital care; Disease may be progressing rapidly	30	Severely disabled; hospital admission is indicated, although death is not imminent.
Emergency care. Pain and infection control	20	Very sick; hospital admission necessary; active, supportive treatment necessary
No treatment	10	Moribund, fatal processes progressing rapidly.
	0	Dead

Katz index activities of daily living

Katz ADL index is the most appropriate instrument for assessing functional status as a measurement of the client's ability to perform activities of daily living independently. The target group is the elderly and people with chronic illnesses. It ranks in six different functions: bathing, dressing, toileting, transferring, continence, and feeding. It scores 1 for a "yes" and 0 for a "No." A score of six indicates full function, four indicates moderate impairment, and two or less indicates severe functional impairment. A person with good knowledge of the client makes the rating.[3, 4]

Groningen Frailty Indicator (GFI)

The Groningen Frailty Indicator (GFI) is a widely employed frailty assessment tool originating from the Netherlands. The GFI comprises fifteen dichotomous self-reported items demonstrating moderate internal consistency and adequate discriminative ability. These items encompass physical factors (independence in shopping, walking, dressing, toileting, physical fitness, vision, hearing, weight loss and polypharmacy), a cognitive component (memory issues), social factors (emptiness, missing others, feeling abandoned), and a psychological component (feeling downhearted or sad; feeling nervous or anxious).

Frailty, as defined by the GFI, is classified on a spectrum ranging from a score of 0 (regular activity without restriction) to 15 (complete disability). A score of 4 or higher indicates frailty. The GFI has been shown to possess favourable feasibility and reliability as a frailty measurement tool. It has been suggested that it can be used in conjunction with the Frailty Index (FI) as a two-step screening process: Initially employing the FI extracted from healthcare data, followed by referral to the GFI questionnaire for patients with a high FI score.

While the majority of GFI studies have been conducted in the Netherlands, cross-cultural validation studies are essential to establish its applicability in diverse populations. [5]

Comprehensive Geriatric assessment (CGA)

This assessment method is a determination process for quantifying and identifying frailty. Here, you examine some risk domains and physical functions. It is a cross-disciplinary diagnostic and treatment process that tries to identify medical, functional, or psychosocial limitations, and in that way, it has a relationship to frailty. [6]

Barthel Index (BI)

The Barthel Index measures the ability to perform ADLs on a ten-item scale, examining functions such as feeding, bathing, grooming, bowel and bladder control, toileting, chair transfer, ambulation, mobility and dressing. It is a robust and widely used functional assessment scale. It can assess linear trajectories and can, therefore, be a useful measure of the progression of frailty over time. As with other frailty tools, it cannot distinguish between causes of functional deterioration. [7]

References

1. Church, S., et al., *A scoping review of the Clinical Frailty Scale.* BMC Geriatr, 2020. **20**(1): p. 393.
2. Friendlander, A.H. and R.L. Ettinger, *Karnofsky performance status scale.* Spec Care Dentist, 2009. **29**(4): p. 147-8.
3. Hartigan, I., *A comparative review of the Katz ADL and the Barthel Index in assessing the activities of daily living of older people.* Int J Older People Nurs, 2007. **2**(3): p. 204-12.
4. Wallace, M. and M. Shelkey, *Katz Index of Independence in Activities of Daily Living (ADL).* Urol Nurs, 2007. **27**(1): p. 93-4.
5. Dent, E., P. Kowal, and E.O. Hoogendijk, *Frailty measurement in research and clinical practice: A review.* Eur J Intern Med, 2016. **31**: p. 3-10.
6. Lee, H., E. Lee, and I.Y. Jang, *Frailty and Comprehensive Geriatric Assessment.* J Korean Med Sci, 2020. **35**(3): p. e16.
7. Chokshi, N.B.K., et al., *A Systematic Review of Frailty Scores Used in Heart Failure Patients.* Heart Lung Circ, 2023. **32**(4): p. 441-453.

Additional file 3 Quality of Life

EuroQol-5D

The EQ-5D is a standard measure for health status introduced by the EuroQol Group. The group's inaugural meeting in 1987 laid the foundation for developing this robust instrument. It is widely used worldwide and has been translated into most major languages through a rigorous translation process[1]. There are three versions of the instrument: EQ-5D-5L, EQ-5D-3L and EQ-5D-Y.
D describes dimensions, and L describes levels.
Each EQ-5D instrument consists of a brief, cognitively undemanding questionnaire that can be completed within minutes. It is designed for self-reporting, but it is also possible to use a proxy version if self-reporting is not possible.
The 3-level version of EQ-5D consists of 2 pages: The EQ-5D descriptive system and the EQ visual analogue scale (EQ-VAS).
The descriptive system comprises five dimensions, each delineating a distinct aspect of health: Mobility, self-care, usual activities, pain/discomfort and anxiety/depression.
Each level has three levels: No-, some- and extreme problems. The patient is asked to indicate their health state by ticking the box next to the most appropriate statement in each of the five dimensions. This decision results in a 1-digit number that expresses the level selected for that dimension. The digits for the five dimensions can be combined into a 5-digit number that describes the patient’s health state.
The EQ-VAS records the patient’s health on a vertical visual analogue scale where the endpoints are labelled best- or worst imaginable health state. It can be used as a quantitative measure of health outcomes that reflects the patient’s judgement.
Example:
Under each heading, patients tick the one box that best describes their health TODAY:
Mobility:
1 I have no problems in walking about
2 I have some problems in walking about
3 I am confined to bed
Self-care
1 I have no problems with self-care
2 I have some problems washing or dressing myself
3 I am unable to wash or dress myself

Usual activities
1 I have no problems with performing my usual activities
2 I have some problems with performing my usual activities
3 I am unable to perform my usual activities

Pain/ Discomfort
1 I have no pain or discomfort
2 I have moderate pain or discomfort
3 I have extreme pain or discomfort

Anxiety/ Depression
1 I am not anxious or depressed
2 I am moderately anxious or depressed
3 I am extremely anxious or depressed

The VAS scale is a visual measure as a ruler between 0 and 100 with the following questions: examining how good or bad their health is TODAY.
100 means the best health you can imagine
0 means the worst health you can imagine
They mark an X on the scale to indicate how their health is TODAY.

SF-36: 36- item Short Form Survey

A widely utilised instrument for assessing health-related quality of life (HRQoL). It is a comprehensive measure of both mental and physical HRQoL. This survey comprehensively evaluates mental and physical dimensions of health-related quality of life (HRQoL). It comprises 36 questions organised into eight distinct domains, collectively contributing to two primary component summaries: the Physical Component Summary (PCS) and the Mental Component Summary (MCS) [2, 3].

The SF-36 questionnaire encompasses eight domains that assess various aspects of health-related quality of life. These domains are as follows:

1. **Physical Functioning** – measures the level of physical activity and the ability to perform daily tasks.
2. **Role Limitations due to Physical Health** – assesses how physical health issues affect work life and daily responsibilities.
3. **Pain** – evaluates the intensity of pain and its impact on everyday activities.
4. **General Health Perceptions** – considers overall self-assessment of health.
5. **Energy/Vitality** – measures levels of energy and fatigue.
6. **Social Functioning** – assesses the ability to engage in social activities without restrictions due to physical or mental health.
7. **Role Limitations due to Emotional Problems** – evaluates the impact of emotional difficulties on work life and daily tasks.
8. **Mental Health** – measures emotional well-being, including depression and anxiety.

For each question, they must choose one option (yes/no, 1 to 5, etc.)
1. In general, would you say your health is good?
2. Compared to one year ago, how would you rate your health in general now?
The following items are about activities you might do during a typical day. Does your health now limit you in these activities? If so, how much?
3. Vigorous activities, such as running, lifting heavy objects, participating in strenuous sports
4. Moderate activities, such as moving a table, pushing a

vacuum cleaner, bowling, or playing golf
5. Lifting or carrying groceries
6. Climbing several flights of stairs
7. Climbing one flight of stairs
8. Bending, kneeling, or stooping
9. Walking more than a mile
10. Walking several blocks
11. Walking one block
12. Bathing or dressing yourself
13. Cut down the amount of time you spend on work or other activities
14. Accomplished less than you would like
15. Were limited in the kind of work or other activities
16. Had difficulty performing the work or other activities

During the past 4 weeks, have you had any of the following problems with your work or other regular daily activities due to any emotional issues?
17. Cut down the amount of time you spend on work or other activities
18. Accomplished less than you would like
19. Did not do work or other activities as carefully as usual
20. During the past 4 weeks, to what extent have your physical health or emotional problems interfered with your everyday social activities with family, friends, neighbours, or groups?
21. How much bodily pain have you had during the past 4 weeks?
22. During the past 4 weeks, how much pain interfered with your everyday work?

The following are about the last 4 weeks.
23. Did you feel full of pep?
24 Have you been a very nervous person?
25. Have you felt so down in the dumps that nothing could cheer you up?
26. Have you felt calm and peaceful?
27. Did you have a lot of energy?
28. Have you felt downhearted and blue?
29. Did you feel worn out?
30. Have you been a happy person?
31. Did you feel tired?
32. During the past 4 weeks, how much of the time have your physical health or emotional problems interfered with your social activities?

How true or false is each of the following statements for you?
33. I seem to get sick a little easier than other people
34. I am as healthy as anybody I know
35. I expect my health to get worse
36. My health is excellent.

SF-12: 12-Item Short Form Survey

It is a concise, shorter form of SF-36. This survey contains 12 items – shown below - that capture essential aspects of SF-36, allowing for a comprehensive, efficient evaluation that is easier and quicker to fill out. It also generates both a physical and a mental component score (PCS and MCS). The survey asks for views about how you feel and how you are able to do normal actives.

SF-12 Health Survey

1. **In general, would you say your health is:**

   Excellent   Very good   Good   Fair   Poor

The following questions are about activities you might do during a typical day. Does your health now limit you in these activities? If so, how much?

2. **Moderate activities such as moving a table, pushing a vacuum cleaner, bowling, or playing golf**
   Yes, limited a lot yes, limited a little   No, not limited at all.

3. **Climbing several flights of stairs**
   Yes, limited a lot yes, limited a little   No, not limited at all

During the past 4 weeks, have you had any of the following problems with your work or other regular daily activities due to your physical health?

4. **Accomplished less than you would like   Yes   No**

5. **Were you limited as a result of your physical health?   Yes   No**

During the past 4 weeks, have you had any of the following problems with your work or other regular daily activities because of any emotional issues (such as feeling depressed or anxious)?

6. **Accomplished less than you would like   Yes   No**

7. **Did work or activities less carefully than usual   Yes   No**

8. **During the past 3 weeks, how much pain has interfered with your normal work (including work outside the home and housework)?**
   Not at all   A little bit   Moderately   Quite a bit   Extremely

These questions are about how you have felt during the past 4 weeks. For each question, please give the one answer closest to how you have been feeling.

How much of the time during the past 4 weeks…

9. **Have you felt calm & peaceful?**
   All of the time.   Most of the time.   A good bit of the time.   Some of the time.   A little of the time.   Never.

10. **Did you have a lot of energy?**
    All of the time.   Most of the time.   A good bit of the time.   Some of the time.   A little of the time.   Never.

11. **Have you felt down-hearted and blue?**
    All of the time.   Most of the time.   A good bit of the time.   Some of the time.   A little of the time.   Never.

12. **During the past 4 weeks, how much of the time has your physical health or emotional problems interfered with your social activities (like visiting friends, relatives, etc.)?**
    All of the time.   Most of the time.   A good bit of the time.   Some of the time.   A little of the time.   Never.

Patient name   Date   PCS   MCS
